# Is dopaminergic medication dose associated with self-reported bruxism in Parkinson’s disease? A cross-sectional, questionnaire-based study

**DOI:** 10.1007/s00784-020-03566-0

**Published:** 2020-09-12

**Authors:** M. C. Verhoeff, M. Koutris, M. K. A. van Selms, A. N. Brandwijk, M. S. Heres, H. W. Berendse, K. D. van Dijk, F. Lobbezoo

**Affiliations:** 1 Department of Orofacial pain and Dysfunction, Academic Centre for Dentistry Amsterdam (ACTA), University of Amsterdam and Vrije Universiteit Amsterdam, Amsterdam, The Netherlands; 2 Amsterdam University Medical Centres (Amsterdam UMC), Neurology, Amsterdam Neuroscience, Vrije Universiteit Amsterdam, De Boelelaan 1117, 1081 HV Amsterdam, The Netherlands; 3 Sleep Wake Centre, Stichting Epilepsie Instellingen Nederland (SEIN), Heemstede, The Netherlands

**Keywords:** Parkinson’s disease, Sleep bruxism, Awake bruxism, Temporomandibular disorders, Dopaminergic medication, Levodopa, Tooth wear

## Abstract

**Objectives:**

It is not clear whether dopaminergic medication influences bruxism behaviour in patients with Parkinson’s disease (PD). Therefore, the aims are to investigate (i) the prevalence of possible (i.e., self-reported) bruxism (sleep and awake) in PD patients, and (ii) whether the use of dopaminergic medication and other factors (viz., demographic characteristics, PD-related factors, and possible consequences of bruxism) are associated with possible bruxism (sleep or awake).

**Materials and methods:**

This study concerns a secondary analysis of an earlier published study. Three hundred ninety-five PD patients (67.9 ± 8.6 years of age; 58.7% males) were included. The levodopa equivalent daily dosage (LEDD) was used as a measure of the dopaminergic medication level. Subsequently, a logistic regression analysis was performed for the dependent variables ‘awake bruxism’ and ‘sleep bruxism’, with the following predictors: gender, age, LEDD, time since PD diagnosis, temporomandibular disorder (TMD) pain, jaw locks, and tooth wear.

**Results:**

The prevalence of possible awake and sleep bruxism was 46.0% and 24.3%, respectively. Awake bruxism was associated with sleep bruxism (OR = 8.52; 95% CI 3.56–20.40), TMD pain (OR = 4.51; 95% CI 2.31–8.79), and tooth wear (OR = 1.87; 95% CI 1.02–3.43). Sleep bruxism was associated with tooth wear (OR = 12.49; 95% CI 4.97–31.38) and awake bruxism (OR = 9.48; 95% CI 4.24–21.19). Dopaminergic medication dose was not associated with awake bruxism (OR = 1.0; 95% CI 0.99–1.00) or sleep bruxism (OR = 1.0; 95% CI 0.99–1.00).

**Conclusion:**

Bruxism is a common condition in PD patients, but is not associated with the dopaminergic medication dose.

**Clinical relevance:**

(Oral) health care providers should be alerted about the possibility of sleep and awake bruxism activity in PD patients, along with this activity’s possible negative health outcomes (viz., TMD pain, tooth wear).

## Introduction

Parkinson’s disease (PD) is a neurodegenerative disease that is characterised by a combination of motor and non-motor symptoms [1]. The classical motor symptoms are bradykinesia, rigidity, and tremor. Examples of non-motor symptoms are cognitive decline, pain, and sleep problems. The aetiology of PD is not fully understood, although it is known that degeneration of dopaminergic neurons in the substantia nigra causes deficits in dopamine levels [1]. The prevalence of PD in The Netherlands is registered at 2 per 1000 persons and is expected to rise [2, 3]. A cure is not yet available. However, suppression of the symptoms through the administration of dopaminergic replacement therapy is possible. Levodopa, the precursor of dopamine, is commonly used for the medical management of PD symptoms [4].

Bruxism is a repetitive jaw-muscle activity characterised by clenching or grinding of the teeth and/or by bracing or thrusting of the mandible. It can occur during sleep (i.e., sleep bruxism) or during wakefulness (i.e., awake bruxism) [5]. The prevalence in the Dutch population is estimated, by means of self-report, at 16.5% and 5% for sleep bruxism and awake bruxism, respectively [6]. The aetiology of bruxism is multifactorial (viz., biological, psychological, and exogenous factors) [7], and treatment is only necessary when negative health outcomes occur [8]. Because diagnosing bruxism behaviour is hard and time-consuming, consensus was reached in which the probability that the behaviour is actually present is graded based on the applied assessment tools. In the present study, self-report was used, and therefore, a ‘possible’ bruxism diagnosis can be established [5].

In patients with bruxism, a side-to-side (right and left hemisphere) imbalance in the dopaminergic system plays a role, especially of the striatal D2 binding potential [9]. When dopaminergic medication is used, this side-to-side imbalance could be reversed in part or completely. Consequently, the number of bruxism episodes is expected to decrease by using levodopa [10]. The relation between medication usage and bruxism in otherwise healthy individuals has been studied before [11, 12]. However, the results of studies on the effect of dopaminergic medication on bruxism are contradictory, some reporting decreases [10, 13, 14], others increases [15], and yet others no effect [12, 16, 17].

Whether bruxism activity in PD is caused by the underlying disease process or the use of dopaminergic medication remains unclear. However, it is clear that both bruxism and PD have their origin in the central nervous system and are both influenced by the dopaminergic nigrostriatal system. Therefore, in PD patients, a possible association between awake bruxism/sleep bruxism and dopaminergic medication can be hypothesised [18]. Based on this premise, the aims of this study are (1) to investigate the prevalence of possible (i.e., self-reported) sleep and awake bruxism in a population of PD patients, and (2) determine whether possible sleep or awake bruxism is associated with the use of dopaminergic medication and/or other factors (viz., demographic characteristics, PD-related factors, and possible consequences of bruxism).

## Material and methods

This study concerns a secondary analysis of a published pilot study of Verhoeff et al. [18], where the association between PD on the one hand, and self-reported bruxism and temporomandibular disorder pain (TMD pain) on the other hand, was analysed [18].

### Data collection

In short, from February 8, 2016, until February 8, 2017, data was collected through a questionnaire. People with and without PD were asked to participate in the study. Questionnaires were distributed at unofficial gatherings of the Dutch association of PD patients (Parkinson Cafés) and social media (viz., Facebook and the website of the Dutch association of PD patients). Only participants with PD were included in the current secondary data analysis. In total, 395 participants with PD filled out the questionnaire.

### Dependent variables

To detect possible (i.e., self-reported) awake and sleep bruxism, the Oral Behaviours Checklist (OBC) was used [19]:

- Possible awake bruxism: ‘do you clench your teeth during waking hours?’, ‘do you press, touch, or hold your teeth together other than while eating (that is, contact between upper and lower teeth)?’, and ‘do you hold, tighten, or tense muscles without clenching or bringing teeth together?’ (responses on a 5-point Likert scale: ‘none of the time’ is scored as 0, ‘all of the time’ is scored as 4). When participants answered more than or equal to ‘sometimes (score 2)’, possible awake bruxism was recorded as being present.
- Possible sleep bruxism: ‘Do you clench or grind your teeth when asleep, based on any information you may have?’ (response on a 5-point Likert scale: ‘none of the time’ is scored as 0, ‘4–7 nights/week’ is scored as 4). When participants answered more than or equal to ‘1–3 nights/month (score 2)’, possible sleep bruxism was recorded as being present.

### Independent variables

Patients were asked to fill in demographic details, general questions about PD, and possible consequences of bruxism (viz., presence of TMD pain, jaw locks, and tooth wear).

- Demographics: ‘what is your gender?’ (male/female) and ‘what is your age?’ (in years).
- Time since PD diagnosis: ‘how long ago where you diagnosed with PD?’ (in years).
- Dopaminergic medication: ‘what medication do you use? Please, fill in the type and dosage per day.’ (mg/day).
- TMD pain: ‘Have you ever had pain in your jaw, temple, in the ear, or in front of the ear on either side?’ (yes/no) [20].
- Jaw locks: ‘Have you ever had your jaw lock or catch, even for a moment, so that it would not open all the way?’ (yes/no)[20].
- Tooth wear: ‘do you have tooth wear?’ (response on a 5-point Likert scale: ‘no tooth wear’ is scored as 0, ‘much tooth wear’ is scored as 4) [21]. A binary outcome is made wherein a score ≥ 1 was scored as the presence of subjective tooth wear.

### Data analysis

Aggregation of medication usage per participant was achieved through the use of the levodopa equivalent daily dosage (LEDD) [22, 23]. According to Tomlinson, the LEDD is a ‘summation of the calculated conversion factors of each individual antiparkinsonian drug, aligned to 100 mg immediate release levodopa’ [22] (see Table 1 for an example). All LEDD scores were calculated by two independent examiners (NB and MH). When no consensus was reached between the two examiners (*N* = 169), a third examiner (MV) calculated the LEDD separately. When consensus was reached, this LEDD score was used. When no consensus was reached or doubt occurred between the three examiners, a neurologist experienced with calculating LEDD scores was contacted (KvD) (*N* = 35). Most of the time, a conflict occurred because participants did not report that a medicine with slow release was used instead of immediate release. However, based on the dosage and frequency of the intake, it could be determined if immediate release or slow release was taken. Also, handwriting mistakes were made (e.g., 2,75 mg instead of 275 mg a day). The following general agreement was made: when medication usage was ambiguous or the medication list was not completed, participants were excluded from the data analysis (*N* = 166). Imputation methods were not used because of the amount of missings (> 20%) and the non-random distribution of the missings, which is in line with the recommendations when to use imputation methods [24].

**Table 1:** An example of a medication list, aligned to 100 mg of immediate release levodopa with the use of conversion factors of different types of dopaminergic medication

Dopaminergic medication	Total daily dose (mg)	Conversion factor	Subtotal LEDD (mg)
Immediate release levodopa	300	1	300
Levodopa slow release	400	0.75	300
Ropinirole	-	20	-
Pramipexole	-	100	-
Pergolide	-	100	-
Bromocriptine	-	10	-
Rotigotine	4	30	120
Amantadine	-	1	-
Apomorphine	-	10	-
Selegiline or rasagiline	-	Total amount levodopa dosage × 0.1	-
COMT inhibitors	800	Total amount levodopa dosage × 0.2	144 (720 × 0.2)
Total LEDD			864

Descriptives were calculated for gender, age, time since PD diagnosis, and LEDD. Besides, the prevalence was calculated for awake bruxism and sleep bruxism. Additionally, for the dependent variables awake bruxism and sleep bruxism, multiple logistic regression models were built and odds ratios with confidence intervals were calculated. First, the unadjusted associations with gender, age, time since PD diagnosis, LEDD, TMD pain, jaw locks, and self-reported tooth wear were determined. Variables that showed at least a weak association (*p* < 0.10) with the outcome variables ‘awake bruxism’ or ‘sleep bruxism’ were included in the multiple logistic regression models. Through the step-by-step approach, the individual variables with the weakest association with the dependent variable were removed from the model (p-to-exit value), until all independent variables showed at least a *p* value < 0.05 in the final model. ORs smaller than 1.5 and ORs above 5 were considered as small and large clinical effect sizes, respectively [25]. All analyses were performed using the IBM SPSS Statistics 26 software package (IBM Corp, Armonk, NY, USA). Probability levels of less than 0.05 were considered statistically significant.

## Results

In Table 2, the demographic characteristics of the participants are presented. The prevalences of possible awake bruxism and sleep bruxism in patients with PD were 46.0% and 24.3%, respectively (see Table 2).

**Table 2:** Demographic information and prevalences of the independent variables (including missings) of the included participants with PD (N = 395)

			Missings (*N*)
Gender (N (%))	Male	232 (58.7%)	0
	Female	163 (41.3%)	0
Age (M, SD)		67.9, SD ± 8.6	4
Time since PD diagnosis (M, SD)		6.7, SD ± 5.9	77
Dopaminergic medication dose, LEDD (M, SD)		710.8, SD ± 469.8	166
Sleep bruxism (*N* (%))		84 (24.3%)	49
Awake bruxism (*N* (%))		161 (46.0%)	45
TMD pain (*N* (%))		112 (29.5%)	15
Locks (*N* (%))		46 (12.3%)	21
Tooth wear (*N* (%))		151 (47.6%)	78

*N*, number of participants; *M*, mean; *SD*, standard deviation

In this study, the LEDD appeared not to be associated with awake or sleep bruxism (see Tables 3 and 4). The results of the single and multiple logistic regression analyses for possible awake bruxism and sleep bruxism are shown in Tables 3 and 4, respectively. The unadjusted associations for awake bruxism showed a possible association (*p* < 0.10) with age (odds ratio (OR) 0.94; 95% CI 0.92–0.97), sleep bruxism (OR 11.50; 95% CI 6.02–21.99), TMD pain (OR 6.78; 95% CI 3.97–11.58), jaw locks (OR 3.83; 95% CI 1.91–7.71), and tooth wear (OR 4.98; 95% CI 3.03–8.17). In the multiple regression analysis, only sleep bruxism (OR 8.82; 95% CI 3.56–20.40), TMD pain (OR 4.51; 95% CI 2.31–8.79), and tooth wear (OR 1.87; 95% CI 1.02–3.43) remained significant. The unadjusted associations for sleep bruxism showed a possible association (*p* < 0.10) with female gender (OR 2.24; 95% CI 1.36–3.68), age (OR 0.94; 95% CI 0.91–0.96), awake bruxism (OR 11.50; 95% CI 6.02–21.99), TMD pain (OR 4.65; 95% CI 2.74–7.88), jaw locks (OR 3.81; 95% CI 1.96–7.41), and tooth wear (OR 16.64; 95% CI 7.26–38.13). According to the multiple regression model, the following variables were significantly associated with the report of sleep bruxism: awake bruxism (OR 9.48; 95% CI 4.24–21.19) and tooth wear (OR 12.49; 95% CI 4.97–31.38). Besides, a trend towards a significant association of sleep bruxism with TMD pain was shown (p-to-exit value 0.057). For both awake and sleep bruxism models, no statistically significant difference was found between the observed and predicted probabilities, according to the Hosmer and Lemeshow test (*p* = 0.92 and 0.85, respectively), concluding that both models fit the observed data.

**Table 3:** Single and multiple regression analysis of variables associated with possible awake bruxism in patients with PD (*N* = 281). The associated *p* value and odds ratio (OR) with 95% confidence interval (CI) are presented

Independent variable	Single regression		Multiple regression		
	*p* value	Odds ratio (95% CI)	p-to-exit value	*p* value	Odds ratio (95% CI)
Gender	0.422	1.19 (0.78–1.83)			
Age	*p* < 0.001	0.94 (0.92–0.97)	0.103		
LEDD	0.736	1.00 (0.99–1.00)			
Time since PD diagnosis	0.161	0.97 (0.93–1.01)			
Sleep bruxism	*p* < 0.001	11.50 (6.02–21.99)		*p < 0.001*	*8.52 (3.56–20.40)*
TMD pain	*p* < 0.001	6.78 (3.97–11.58)		*p < 0.001*	*4.51 (2.31–8.79)*
Locks	*p* < 0.001	3.83 (1.91–7.71)	0.111		
Tooth wear	*p* < 0.001	4.98 (3.03–8.17)		*0.044*	*1.87 (1.02–3.43)*

*R*^2^ = .394 (Nagelkerke), 0.294 (Cox and Snell). *X*^2^ (3) = 97.8, *p* < 0.001

**Table 4:** Single and multiple regression analysis of variables associated with possible sleep bruxism in patients with PD (*N* = 283). The associated *p* value and odds ratio (OR) with 95% confidence interval (CI) are presented

Independent variable	Single regression		Multiple regression		
	*p* value	Odds ratio (95% CI)	p-to-exit value	*p* value	Odds ratio (95% CI)
Gender	0.002	2.24 (1.36–3.68)	0.124		
Age	*p* < 0.001	0.94 (0.91–0.96)	0.394		
LEDD	0.834	1.00 (0.99–1.00)			
Time since PD diagnosis	0.551	1.01 (0.97–1.06)			
Awake bruxism	*p* < 0.001	11.50 (6.02–21.99)		*p < 0.001*	*9.48 (4.24–21.19)*
TMD pain	*p* < 0.000	4.65 (2.74–7.88)	0.057		
Locks	*p* < 0.001	3.81 (1.96–7.41)	0.484		
Tooth wear	*p* < 0.001	16.64 (7.26–38.13)		*p < 0.001*	*12.49 (4.97–31.38)*

*R*^2^ = .48(Nagelkerke), 0.32 (Cox and Snell). *X*^2^ (2) = 109.5, *p* < 0.001

## Discussion

The first aim of the present study was to determine the prevalence of possible awake bruxism and sleep bruxism in a population of PD patients. The results showed a respective prevalence of 46.0% and 24.3% for these conditions. The second aim was to investigate possible associations between the dose of dopaminergic medication and the presence of awake and sleep bruxism. The results showed that in a PD population, the levodopa equivalent daily dosage (LEDD) was not associated with the self-reports of awake and sleep bruxism. Hence, the hypothesis formulated in the introduction, viz., that there is an association between awake bruxism/sleep bruxism and dopaminergic medication, could not be accepted. Furthermore, this study examined whether other factors were significantly associated with self-reported awake and sleep bruxism. Co-occurrence of both awake bruxism and sleep bruxism was observed. Besides, there was an association with tooth wear and both circadian manifestations of bruxism. Finally, awake bruxism was also found to be associated with TMD pain.

### Prevalence of awake and sleep bruxism

In the studied population, awake bruxism and sleep bruxism were reported much more often (46% and 24.3%, respectively) than in the general population of the same age (3% and 8.3%, respectively) [6]. While this suggests a large discrepancy with the results of our study, as earlier stated in the hypothesis, it is not that surprising. It is known that populations with neurological conditions show a higher prevalence of awake bruxism [26]. Moreover, some risk factors for bruxism are more prevalent in patients with PD [27]. Examples of risk factors for bruxism are, amongst others, the presence of stress and depressive thoughts [28–31] and the use of specific types of medication, such as selective serotonin reuptake inhibitors (SSRI) [11, 12]. All of these risk factors are more prevalent in patients with PD than in the general population [27]. Finally, it has been demonstrated that patients with PD have an increased prevalence of sleep problems, resulting in an increased occurrence of arousals from sleep which is in turn related to higher numbers of sleep bruxism events [32, 33].

### Bruxism and dopaminergic medication

The present study is the first to analyse the association between dopaminergic medication dose and bruxism using LEDD scores in a PD population. As indicated in the Introduction, dopaminergic medication can have variable effects on bruxism in otherwise healthy individuals [10, 13–17]. In the present study, however, PD patients were included, which makes it difficult, if not impossible, to compare the present findings to the previously reported ones [10, 13–17]. Only in the case report described by Magee (1970) [15], levodopa usage in a PD patient was reported. In that patient, use of levodopa resulted in the occurrence of bruxism behaviour [15]. However, case reports do not provide solid scientific evidence for the described observations. Consequently, the present findings cannot be compared with the study of Magee (1970) either [15]. In sleep laboratory studies, it was shown in healthy volunteers that levodopa exerts an attenuating effect on sleep bruxism [10]. For the usage of bromocriptine, a dopamine agonist, conflicting results were shown. On the one hand, Lobbezoo et al. showed a reduction of sleep bruxism [13], while on the other hand, Lavigne et al. showed that bromocriptine did not reduce or exacerbate sleep bruxism [16]. Finally, Cahlin et al. found that pramipexole, also a dopamine agonist, had no attenuating effect on sleep bruxism [17]. Future studies should be performed in PD patients in whom definite diagnoses of bruxism have been established, as opposed to the possible diagnoses set in the present study. Such studies could also shed further light on the question whether bruxism in PD patients is medication-dependent or rather associated with the neurodegenerative disease itself.

Pharmacokinetics could have played a role in the present results. Dopaminergic medication can act on different types of dopamine receptors. These receptors can have decreasing or increasing effects on dopamine levels [34]. Therefore, it is possible that different drugs can either worsen or ameliorate bruxism, depending on the specific working mechanisms. Hence, different effects on bruxism can occur when analysing the LEDD in total or for each prescription drug individually. However, it is not desirable to ignore the coherence of the subscribed drugs in this specific population. Therefore, the total LEDD score was calculated per participant.

Different studies showed different mean LEDD scores, varying from 804 mg (SD ± 364) to 1409 mg (SD ± 605) [35–38]. These differences are possibly due to geographical differences or differences in disease stage. In the present study, the mean LEDD score was 710.8 (SD ± 469.8). This relatively low mean score is possibly due to the low mean time since PD diagnosis in the present study: 6.7 (SD ± 5.9) years. This low mean LEDD score might implicate that PD symptoms and dopaminergic medication usage were not likely to cause pronounced side effects compared with patients who have been diagnosed with PD a long time ago and/or have higher LEDD values. Besides, when chronic depression of dopamine is present, high doses are required to achieve symptom relief. These enhanced maladaptive changes could lead to levodopa-induced dyskinesia. When this appears in the orofacial area, it can be confused with bruxism and vice versa [39]. The mix-up with orofacial levodopa-induced dyskinesia and the low LEDD scores could have led to the rejection of the hypothesis of the present study. In future studies, patients with a longer duration of PD, and thus a probably longer use of dopaminergic medication, should be included. However, such individuals are probably not capable of visiting Parkinson Cafés and/or do not use social media, and are for that reason probably not included in the present study.

### Bruxism and other associated factors

Several studies have shown an association between both circadian manifestations of bruxism and TMD pain. However, also contradictory results exist [40, 41]. In the present study, it was found that in this PD population, awake bruxism was significantly associated with TMD pain, while sleep bruxism only showed a trend towards an association with TMD pain. This difference can be due to the fact that in the present study, awake bruxism was reported almost 50% more often than sleep bruxism. The association between (awake) bruxism and TMD pain in this population could be explained by the fact that the load-bearing capacity (i.e., the physical capacity of individuals to endure muscle-induced load on the structures of the masticatory system) of patients with PD can be reduced, which can result in TMD pain. Besides, pain, in general, is a common non-motor symptom that is present in PD and can also be present in the orofacial area [42]. PD patients can experience different types of pain, such as musculoskeletal pain (40–75%), but also neuropathic pain. The latter can imply that pain processing in PD patients, in the peripheral or central nervous system, can be amplified [42]. However, in 6-OHDA-treated rats (i.e., rats used as PD model), bilateral mechanical hypernociception showed a reduction when undergoing dopaminergic therapy [43]. This could implicate that dopaminergic therapy could reduce nociception and therefore pain perception in patients with PD.

While bruxism during wakefulness and bruxism during sleep are commonly considered two separate entities, in the present study, a co-occurrence between awake and sleep bruxism was observed. It is noteworthy that even based on the current definition of generic bruxism there is a clear distinction between awake and sleep bruxism, based on the assumption that both conditions do not share the exact same pathophysiology [5]. Nevertheless, there are, for example, psychosocial aspects related to both awake and sleep bruxism that may explain their association [44]. Therefore, the possibility of a co-existence between both circadian manifestations must be taken into account when interpreting awake and sleep bruxism in the future.

Both awake and sleep bruxism showed an association with self-reported tooth wear. The predicted prevalence of severe tooth wear according to different studies ranged between 12 and 17% in participants of 65–70 years of age [45, 46]. A recently published narrative overview described that tooth wear is associated with sleep bruxism; not with awake bruxism [47]. This can be explained considering that during wakefulness, clenching occurs more frequently than tooth grinding, which can result in less tooth wear. The difference with the general population is the higher prevalence of both sleep bruxism and awake bruxism in PD patients. Besides, there is a possibility that during wakefulness, not only clenching but also tooth grinding is present in patients with PD, due to the involuntary movements. Furthermore, other factors that can influence the amount or severity of tooth wear [47] are also common in patients with PD (viz., polypharmacy and gastro-oesophageal reflux disease) [48, 49].

### Limitations of the study

This study has several limitations, due to which an association could have been missed between LEDD and bruxism. According to the international consensus, based upon self-report, a definite diagnosis of bruxism cannot be established [50]. Hence, in the present study, only a diagnosis of possible bruxism could be established. Consequently, one has to be careful when interpreting the current findings. However, the advantage of self-report is evident, viz., that assessing a larger sample is feasible, as opposed to the usage of instrumental techniques that are required for establishing definite diagnoses of awake and sleep bruxism (viz., electromyography, polysomnography) [5]. In future studies, a multimodal assessment could improve the understanding of bruxism in this population [51]. Concerning tooth wear, the answers given by participants were subjective. Collecting objective tooth wear data in future clinical studies will improve the validity of these results. Furthermore, the questions were formulated in such manner that they lacked time sensitivity regarding complaints in the orofacial area and bruxism behaviour. Therefore, it is possible that these complaints and this behaviour had already evolved before the start of the medication intake, which could have resulted in some false-positive responses. To overcome these limitations, future studies should take time-sensitive aspects into account. Another limitation of the present study is the large number of missing data (42%), because reports of medication usage were frequently ambiguous or incomplete. A possible explanation is the difficulty with writing by hand and/or the amount of work participants experienced while completing the questionnaire. Therefore, the data must be interpreted with caution. Furthermore, selection bias could have been possible due to the location where this study was conducted (Parkinson Cafés and social media). The severity of the disease may therefore be lower than in the overall population of patients with Parkinson’s disease in The Netherlands. Finally, this study concerns a secondary analysis of an earlier published pilot study [18]. In general, a disadvantage of such analysis could be that data is outdated. However, the advantages are clear, viz., cost- and time-efficiency as well as making optimal use of data that were collected from vulnerable participants which could be considered an ethical plus. Besides, this is the first study that calculated the LEDD scores and analysed them in the association with bruxism. Nonetheless, for further research, the authors would like to suggest that the LEDD scores are calculated based on data on medication usage provided by the pharmacist or neurologist for a more reliable outcome and fewer missing values.

## Conclusion

The prevalence of possible (i.e., self-reported) awake bruxism and sleep bruxism in patients with PD was high, viz., 46.0% and 24.3%, respectively. No association was found between dopaminergic medication usage and possible (i.e., self-reported) bruxism. Furthermore, in a population with PD patients, co-occurrence of both circadian manifestations of bruxism is present. Besides, both conditions are associated with self-report of tooth wear. Finally, only possible awake bruxism was found to be associated with TMD pain, not possible sleep bruxism.
